# Characteristics of progressive supranuclear palsy presenting with corticobasal syndrome: a cortical variant

**DOI:** 10.1111/nan.12037

**Published:** 2014-01-21

**Authors:** H Ling, R de Silva, L A Massey, R Courtney, G Hondhamuni, N Bajaj, J Lowe, J L Holton, A Lees, T Revesz

**Affiliations:** * Reta Lila Weston Institute of Neurological Studies, Institute of Neurology, University College LondonLondon, UK; † Queen Square Brain Bank for Neurological Disorders, Institute of Neurology, University College LondonLondon, UK; ‡ Sara Koe PSP Research Centre, Institute of Neurology, University College LondonLondon, UK; § Department of Clinical Neurology, Nottingham University Hospitals NHS TrustNottingham, UK; ¶ Department of Pathology, Queen's Medical Centre, University of NottinghamNottingham, UK

**Keywords:** alien limb, corticobasal syndrome, progressive supranuclear palsy, Richardson's syndrome, tau

## Abstract

**Aims:**

Since the first description of the classical presentation of progressive supranuclear palsy (PSP) in 1963, now known as Richardson's syndrome (PSP-RS), several distinct clinical syndromes have been associated with PSP-tau pathology. Like other neurodegenerative disorders, the severity and distribution of phosphorylated tau pathology are closely associated with the clinical heterogeneity of PSP variants. PSP with corticobasal syndrome presentation (PSP-CBS) was reported to have more tau load in the mid-frontal and inferior-parietal cortices than in PSP-RS. However, it is uncertain if differences exist in the distribution of tau pathology in other brain regions or if the overall tau load is increased in the brains of PSP-CBS.

**Methods:**

We sought to compare the clinical and pathological features of PSP-CBS and PSP-RS including quantitative assessment of tau load in 15 cortical, basal ganglia and cerebellar regions.

**Results:**

In addition to the similar age of onset and disease duration, we demonstrated that the overall severity of tau pathology was the same between PSP-CBS and PSP-RS. We identified that there was a shift of tau burden towards the cortical regions away from the basal ganglia; supporting the notion that PSP-CBS is a ‘cortical’ PSP variant. PSP-CBS also had less severe neuronal loss in the dorsolateral and ventrolateral subregions of the substantia nigra and more severe microglial response in the corticospinal tract than in PSP-RS; however, neuronal loss in subthalamic nucleus was equally severe in both groups.

**Conclusions:**

A better understanding of the factors that influence the selective pathological vulnerability in different PSP variants will provide further insights into the neurodegenerative process underlying tauopathies.

## Introduction

The classical presentation of progressive supranuclear palsy (PSP), now known as Richardson's syndrome (PSP-RS), includes as cardinal features the early onset of postural instability with falls backwards, vertical supranuclear gaze palsy (VSGP) including downgaze and frontal subcortical cognitive impairment 1–3. Other well-recognized clinical variants of PSP are PSP-parkinsonism (PSP-P) 2,4, pure akinesia and gait freezing (PSP-PAGF) 5,6, primary non-fluent aphasia (PSP-PNFA) 7–10, behavioural variant of frontotemporal dementia (PSP-bvFTD) 11 and corticobasal syndrome (PSP-CBS) 12. Clinicopathological studies have since demonstrated a close correlation between topographical severity of tau pathology and clinical phenotypes of PSP. For instance, severe tau pathology was identified in the inferior frontal gyrus in PSP-PNFA 8 and frontal and temporal cortices in PSP-bvFTD 13. In contrast, cortical tau was found to be very mild in the PSP-PAGF subtype 5,6. Similar clinicopathological correlation was also identified in another closely related 4-repeat (4R) tauopathy, corticobasal degeneration (CBD) and its clinical phenotypes 14.

Corticobasal syndrome (CBS) describes progressive clumsiness and loss of function of one hand due to apraxia, an alien limb, cortical sensory loss, dystonia and levodopa-unresponsive rigidity, and it was initially described as the distinctive clinical presentation of CBD 15,16. Since its original description, multifarious other pathologies have been linked to a CBS presentation 7,17,18. From the archives of the Queen Square Brain Bank for Neurological Disorders (QSBB), we showed that the most common underlying pathology for CBS is PSP (6 of 21) rather than CBD (5 of 21); however, only 4% of all pathologically diagnosed PSP cases (*n* = 227) had a CBS presentation (PSP-CBS) 17. Previously, Tsuboi *et al*. quantified tau load in four selected cortical regions including cingulate gyrus, mid-frontal cortex, motor cortex and inferior-parietal cortex in three PSP-CBS cases and eight randomly chosen PSP-RS cases 12. They reported an increased tau pathology in the mid-frontal and inferior-parietal cortices in PSP-CBS compared with PSP-RS and concluded that the CBS presentation of PSP was either caused by a concurrent cortical pathology from a secondary process such as Alzheimer's disease or primary PSP tau pathology involving the cortical regions 12. Nevertheless, it is uncertain if differences exist in the distribution of tau pathology in other brain regions or if the overall tau load is increased in the brains of PSP-CBS. It is noteworthy that imaging studies have identified predominant focal grey matter loss on voxel-based morphometry in premotor cortex, posterior superior frontal lobe and supplementary motor area and relatively preserved brain stem grey matter in cases with PSP-CBS 19. We therefore hypothesized that the distribution of tau pathology in PSP-CBS may resemble the distribution of grey matter loss identified by *in vivo* imaging in voxel-based morphometry.

The aims of this study were: (i) to validate the findings reported by Tsuboi *et al*. in a significantly larger cohort of PSP-CBS cases and to quantitatively assess tau distribution in more cortical regions and other brain regions including the basal ganglia, brainstem and cerebellum; (ii) to determine the cellular lesions which contribute to the tau pathology were characteristic of PSP pathology rather than Alzheimer-type neurofibrillary tangle pathology; and (iii) to assess neuronal loss of the substantia nigra and subthalamic nuclei and pathological involvement of the corticospinal tract.

## Materials and methods

### Cases

Of the 227 PSP cases available in the QSBB archives between 1988 and 2010, nine had received a final clinical diagnosis of CBS/CBD by a neurologist during life (PSP-CBS, 3.9% of all PSP cases). An additional case, seen and diagnosed pathologically at the University of Nottingham, was also included. These 10 PSP-CBS cases were matched with 10 PSP-RS control cases for disease duration and age at death. The brain donor programme of the QSBB was approved by a London Multi-Centre Research Ethics Committee and tissue is stored for research under a license from the Human Tissue Authority.

### Medical record review

Systematic retrospective review of the medical records was carried out by one of us (H. L.). All patients were assessed by at least one neurologist during life. Symptoms and clinical signs were recorded as being absent if they were not reported in the case notes. When the onset was not recorded, the onset was taken as the time when the particular clinical feature was first mentioned in the notes. If there were conflicting clinical features, the findings of the neurologist took precedence. The definitions for each selected clinical feature have been described previously 17.

### Pathological material

The neuropathological diagnosis of PSP was confirmed in all 20 cases (T. R. and J. L. H.). Immediately after *post mortem* the brains were divided in the mid-sagittal plane. One half, chosen randomly, was sliced and tissue blocks were frozen and stored at −80°C, while the other half was immersed and fixed in 10% neutral formalin for 3 weeks before neuropathological examination. Tissue blocks were taken using standard protocols. Established pathological diagnostic criteria for PSP were used, requiring the presence of neurofibrillary tangles (NFTs), neuropil threads (NTs) and glial tau pathology in different brain regions including the cerebral cortex, striatum, globus pallidus (GP), subthalamic nucleus (STN), midbrain, pons and cerebellum together with neuronal loss and gliosis in basal ganglia and brainstem and cerebellar nuclei 20–23. In each case 8-μm-thick tissue sections cut from the paraffin blocks and stained with haematoxylin and eosin (H&E) were used to assess neuronal loss and gliosis in the basal ganglia and substantia nigra. Immunohistochemistry with antibodies to phosphorylated tau (AT8; BioScience Life Sciences; 1:600), 3-repeat (3R) tau and 4R tau (Upstate/Millipore; 3R tau: RD3; 1:2000; 4R tau: RD4; 1:200) 24, microglia (CD68; Dako; PG-M1; 1:75), αB-crystallin (Novocastra; G2JF; 1:300), amyloid-β (Aβ) peptide (Dako; 6F/3D; 1:100) and α-synuclein (Vector Laboratories; KM51; 1:50) was performed using a standard avidin-biotin method as previously described 25.

Additional pathologies were documented. Argyrophilic grain disease was identified by AT8 and αB-crystallin immunohistochemistry 26 while Aβ cortical plaque pathology was characterized using the modified CERAD (Consortium to Establish a Registry for Alzheimer's disease) criteria 27. Alzheimer's type NFT pathology was determined using AT8 immunohistochemistry for Braak and Braak staging 28. The presence of incidental Lewy body disease 29, cerebrovascular disease 30 and cerebral amyloid angiopathy (CAA) 31 was documented. Only cases with limited Alzheimer-type neurofibrillary tangle pathology of Braak and Braak stage III or less were recruited to avoid confounding the analysis of PSP-related tau pathology.

### Regional tau quantification with image analysis

Using coded slides, quantitative assessment of tau pathology, comprising all tau-positive structures including NFTs, pretangles (PreTs) NTs, tufted astrocytes (TAs) and coiled bodies (CBs) was performed by one rater (H. L.). Fifteen brain regions, which are known to be affected in PSP and whose involvement is predicted to contribute to the clinical features, were selected; the posterior frontal cortex including the motor strip, cerebral cortex and subcortical white matter of the middle frontal gyrus (level: 1 cm behind the temporal pole), middle temporal gyrus (level: mammillary body) and parietal region (level: 1 cm behind the splenium), caudate nucleus, putamen, GP, STN, substantia nigra (SN; level: emergence of the third cranial nerve), pontine base, including the pontine nuclei, cerebellar dentate nucleus and cerebellar white matter. The posterior frontal white matter was omitted from the analysis as the quantity was very small in some cases due to variability of routine sampling. In each region, the images of 10 random microscopic fields using a ×20 objective were captured by a colour digital camera connected to the microscope (Nikon Microphot-FXA and Digit sight DS-L1) and processed with an image analysis software (Image-Pro; Media Cybernetics, Inc., Roper Industries, Rockville, USA), converted to grey-scale images and labelling was measured in pixels. Threshold was adjusted to capture the two-dimensional area of all tau-positive lesions and the same threshold setting was used throughout the study. ‘Areal fraction’, defined by a ratio of the tau-positive immunoreactive pixels to the total number of pixels of the whole field was computed by Image Pro and tau load for each region, that is, ‘regional’ tau load was expressed as percentage (areal fraction × 100%) 32. ‘Total’ tau load was the sum of tau load in all 15 regions. ‘Cortical’ tau load was the sum of tau load in seven regions, comprised of both grey and subcortical white matter in the anterior frontal, temporal and parietal regions and grey matter in the posterior frontal region. ‘Basal ganglia’ tau load was the sum of tau load in four structures: caudate nucleus, putamen, GP and STN.

### Quantification of tau-positive cellular lesions

The different tau-positive cellular lesions were quantified individually in 10 random fields of three selected regions (posterior frontal cortex, anterior frontal cortex and caudate), where differences in regional tau load were found to be the most robust between PSP-RS and PSP-CBS. NFTs, PreTs, TAs and CBs were individually counted. NT pathology was quantified using a four-tiered semi-quantitative grading scale (0–3, with grade 0 = no NT to grade 3 = most severe NT).

### Neuronal loss in the subthalamic nucleus and substantia nigra

Neuronal loss in STN and SN were determined using a four-tiered semi-quantitative grading system by a neuropathologist (T. R.), blinded to the clinical features (0–3, with grade 0 = no neuronal loss to grade 3 = most severe neuronal loss). SN was divided into five regions (medial, dorsomedial, dorsolateral, ventrolateral and lateral).

### Corticospinal tract involvement

Microglial pathology of the corticospinal tract (CST) identified in the midbrain cerebral peduncles was assessed using CD68 immunohistochemistry by two neuropathologists (T. R. and J. H.) blinded to the clinical features. A semi-quantitative grade was established by consensus (grade 0 = baseline microglial population to grade 3 = most severe microglial pathology).

### Tau biochemistry

Frontal cerebral cortex was used for tau biochemistry in two PSP-CBS, two PSP-RS cases and two pathologically diagnosed CBD cases with classical CBS presentation (CBD-CBS), which were randomly selected. Regional variation of phosphorylated tau species in PSP brains was previously reported 33. However, tau protein extraction was limited to the frontal cortex in the present study.

#### Sarkosyl-insoluble tau isolation

Isolation of sarkosyl-insoluble tau was carried out as previously described 34,35. Brain tissue was homogenized in 10× volume (v/w) homogenization buffer (10 mM Tris–HCl pH 7.4, 0.8 M NaCl, 1 mM EGTA and 10% sucrose containing Complete protease inhibitor cocktail (Roche, Burgess Hill, UK). The suspension was then spun at 20 000 **g** for 20 min at 4°C and the supernatant set aside. The pellet was re-suspended in 5× volumes of homogenization buffer and re-centrifuged as above. The supernatants were combined and *N-lauryl* sarcosinate added to a concentration of 1% (w/v), and incubated at room temperature for 1 h with shaking. The mixture was then centrifuged at 100 000 **g** for 1 h at 4°C. The sarkosyl-insoluble pellet was re-suspended in 50 mM Tris–HCl pH 7.5 at 0.2 ml/g of starting material.

#### SDS-PAGE

Sarkosyl-insoluble tau was separated on 10% SDS-polyacrylamide gels and blotted onto nitrocellulose membranes using standard procedures. The blots were probed with a pan-tau rabbit polyclonal TP70 antibody that recognizes the carboxy-terminus of tau 36,37 (1/15 000; kind gift from Dr Diane Hanger, King's College, London) and IRDye 800CW Donkey Anti-Rabbit secondary antibody (Li-Cor Biosciences) followed by imaging on a Li-Cor Odyssey Infrared Scanner.

### Haplotype analysis of the *MAPT* gene

Haplotype was determined by PCR (polymerase chain reaction) typing of the 238 bp *MAPT* H2 deletion in intron nine in 17 cases (8 PSP-CBS, 9 PSP-RS) where frozen tissue was available for DNA extraction 38,39.

### Statistical analysis

The Mann–Whitney *U*-Test was used to compare tau load between PSP-CBS and PSP-RS. The null hypothesis (H0) was rejected if the *P* value was <0.05 when ‘total’, ‘cortical’ and ‘basal ganglia’ tau load was assessed. For ‘regional’ tau load assessment, *P* value of 0.0033 (0.05/15) was used to adjust for multiple comparisons; for tau-positive cellular lesion load, *P* value of 0.01 (5 different types of tau lesions: 0.05/5) was used. χ^2^/Fisher's exact test or the Student's *t*-test was used to compare semi-quantitative grading or clinical data using *P* value of 0.05. The intra-rater repeatability was assessed by repeating tau quantification in four randomly selected cases (20%). The intraclass correlation coefficient was 0.80 (*P* < 0.001), indicating that the ‘regional’ tau load results were highly repeatable. The spss 17.0 program (IBM Corporation, New York, USA) was used for statistical analysis.

## Results

### Clinical features

#### PSP-CBS (Tables 1a and 2)

All patients had been diagnosed with CBS/CBD by neurologists during life. Mean duration of first symptom onset to the final clinical diagnosis was 3.4 years. All cases had strikingly asymmetrical clinical features throughout the entire disease course; 10 had ideomotor limb apraxia, eight had hand dystonia, five had focal distal myoclonus, three had an alien limb phenomenon, three had non-fluent aphasia, three had cortical sensory loss and wo had hemisensory neglect. Delayed initiation of horizontal saccades was observed in three patients, two of whom also had head thrust at saccadic initiation (cases 2 & 5).

**1a:** Demographic, clinical and genetic haplotype data of PSP-CBS patients

	PSP-CBS									
Case no.	1	2	3	4	5	6	7	8	9	10
Gender	M	F	M	M	M	F	F	F	M	F
Age at onset (yr)	55	63.8	60.4	60.5	79.3	66.3	60	64	77	73
Age at death (yr)	64.3	70.2	66.3	68.8	82.8	77.9	70.8	72.5	81	79.2
Disease duration (yr)	9.3	6.4	5.9	8.3	3.5	11.6	10.8	8.5	4	6.2
Initial clinical Dx	PD	CBS	C.Spond.	CVD	PD	Depression	CBS	PSP	PSP	CBS
Final clinical Dx	CBS	CBS	CBS	CBS	CBS	CBS	CBS	CBS	CBS	CBS
Duration from onset to final Dx (yr)	6.3	3.5	5.2	2.1	1.5	2.6	7	6	4	3
Initial symptom(s)	Balance difficulty	Clumsy useless arm	Clumsy useless arm	Jerky arm	Falls	Gait difficulty & cognitive slowing	Clumsy useless arm & balance difficulty	Falls	Balance difficulty & slurred speech	Clumsy useless arm & falls
Asymmetrical features	+	+	+	+	+	+	+	+	+	+
Limb apraxia	+	+	+	+	+	+	+	+	+	+
Alien limb	−	+	−	−	−	−	−	−	+	+
Cortical sensory loss	−	+	+	−	−	−	−	−	−	−
Hemi-neglect	−	+	−	−	−	−	−	+	−	−
Aphasia	−	+	−	−	−	+	−	−	+	−
Hand dystonia	+	+	+	+	−	−	+	+	+	+
Clenched fist	+	+	+	+	−	−	+	−	+	+
Myoclonus	−	−	+	+	+	−	+	−	+	−
Tremor	−	−	−	−	+	−	−	−	+	−
Delayed initiation of saccades	NK	+	NK	+	+	NK	NK	−	−	NK
Slow vertical saccades	NK	−	NK	+	+	+	NK	+	NK	+
VSGP	+	−	NK	−	+	+	NK	+	+	−
Postural instability/falls within 1st yr	−	+	−	−	+	+	−	+	+	+
Cognitive decline	−	+	−	+	+	+	−	+	−	+
Personality change/apathy	−	−	−	+	−	+	−	−	−	−
Pyramidal signs	+	+	−	+	+	−	+	−	−	−
Akinetic rigidity in first 2 yrs	+	−	−	−	+	+	−	+	+	+
Dysarthria in first 2 yrs	+	−	−	+	−	−	−	−	+	−
Dysphagia in first 2 yrs	−	−	−	NK	−	−	NK	NK	+	−
Levodopa response	−	−	−	−	Mild	−	−	−	−	Mild
H1/H2 Haplotype	H1/H1	H1/H1	H1/H1	NK	H1/H1	H1/H2	H1/H1	H1/H1	NK	H1/H2

Seven patients developed ocular features suggestive of PSP including slow vertical saccades or VSGP but in most cases these occurred in the advanced stage of the illness. Two exceptions were cases 8 and 9 who developed VSGP within 4 years from symptom onset and an initial clinical diagnosis of PSP was considered, but was later revised to CBS after the onset of asymmetrical cortical symptoms. Six patients developed postural instability or falls within the first year of symptom onset. Nevertheless, VSGP (χ^2^, *P* = 0.016) and postural instability or early falls were still more frequent in PSP-RS than in PSP-CBS. Pyramidal signs were more frequent in PSP-CBS (*n* = 5) than in PSP-RS (*n* = 0) (χ^2^, *P* = 0.016). Extensor plantars and hyper-reflexia were noted in five PSP-CBS patients, three of whom also had spasticity and one had pyramidal weakness, but none of these features was observed in PSP-RS.

#### PSP-RS (Tables 1b and 2)

All PSP-RS patients had a final clinical diagnosis of probable PSP and had VSGP including downgaze abnormalities and early postural instability or falls. Three patients had cognitive decline and five had frontal type personality change characterized by apathy and abulia.

**1b:** Demographic, clinical and genetic haplotype data of PSP-RS patients

	PSP-RS									
Case no.	11	12	13	14	15	16	17	18	19	20
Gender	F	F	M	M	F	M	M	F	F	M
Age at onset (yr)	62	65.2	63	74.3	66	61	52.1	67	72	76
Age at death (yr)	69.8	71.3	69.5	79.5	81.7	78.3	61.3	73	79.1	80.7
Disease duration (yr)	7.8	6.1	6.5	5.2	15.7	17.3	9.2	6	7.1	4.7
Initial clinical Dx	Depression	PD	Depression	PD	PD	CVD	PSP	PSP	PSP	PSP
Final clinical Dx	PSP	PSP	PSP	PSP	PSP	PSP	PSP	PSP	PSP	PSP
Duration from onset to final Dx (yr)	4	4	2.5	3	7	2.2	3	3	3	2
Initial symptom(s)	Falls & cognitive slowing	Falls	Falls & cognitive slowing	Falls	Slow up	Slurred speech	Balance difficulty	Falls	Falls	Falls
Asymmetrical features	−	−	−	−	−	−	−	−	−	−
Limb apraxia	−	−	−	−	−	−	−	−	−	−
Alien limb	−	−	−	−	−	−	−	−	−	−
Cortical sensory loss	−	−	−	−	−	−	−	−	−	−
Hemineglec	−	−	−	−	−	−	−	−	−	−
Aphasia	−	−	−	−	−	−	−	−	−	−
Hand dystonia	−	−	−	−	−	−	+	−	−	−
Clenched fist	−	−	−	−	−	−	−	−	−	−
Myoclonus	−	−	−	−	−	−	−	−	−	−
Tremor	+	−	−	−	−	−	−	−	−	+
Delayed initiation of saccades	NK	−	−	NK	−	−	−	−	−	NK
Slow vertical saccades	+	NK	+	NK	+	+	NK	+	+	NK
VSGP	+	+	+	+	+	+	+	+	+	+
Postural instability/falls within 1st yr	+	+	+	+	+	+	+	+	+	+
Cognitive decline	+	−	+	−	−	−	−	+	−	−
Personality change/apathy	+	−	+	+	−	−	+	+	−	−
Pyramidal signs	−	−	−	−	−	−	−	−	−	−
Early akinetic rigidity in first 2 yrs	+	+	+	+	+	+	+	+	+	+
Early dysarthria in first 2 yrs	+	NK	−	−	−	+	+	NK	+	−
Early dysphagia in first 2 yrs	+	−	−	−	−	−	+	NK	+	−
Levodopa response	−	−	−	−	−	−	−	−	−	−
H1/H2 Haplotype	H1/H1	H1/H1	NK	H1/H1	H1/H1	H1/H1	H1/H1	H1/H1	H1/H1	H1/H1

CBS, corticobasal syndrome; C.Spond, cervical spondylosis; CVD, cerebrovascular disease; Dx, diagnosis; F, female; M, male; NA, not applicable; NK, not known; PD, Parkinson's disease; PSP, progressive supranuclear palsy, RS, Richardson's syndrome; VSGP, vertical supranuclear gaze palsy; yr, year.

**Table 3:** Demographic features between PSP-CBS and PSP-RS

	PSP-CBS	PSP-RS	*P* values (Student's *t*-test)
	(mean years ± SD)		
Mean age of symptom onset	65.9 ± 8.0	65.9 ± 7.1	0.98
Mean age of death	73.4 ± 6.4	74.4 ± 6.5	0.72
Mean disease duration	7.5 ± 2.7	8.6 ± 4.4	0.51

### Pathological findings and clinicopathological correlations

Both PSP-CBS and PSP-RS groups met established pathological criteria of PSP 20–22. All inclusion types were immunoreactive for 4R tau by differential immunohistochemistry but negative for 3R tau in all cases 24, which was an expected finding for PSP.

#### ‘Regional’ tau load

**Figure 1:**
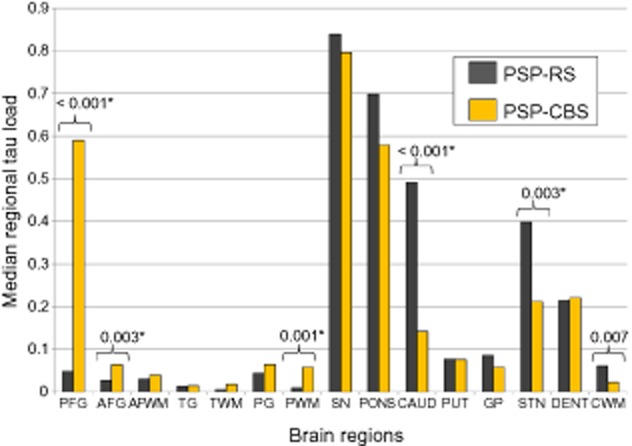
Quantitative data illustrating median regional tau load in PSP-RS (black) and PSP-CBS (yellow) in 15 selected regions. Error bars represent 95% confidence interval. *Represents statistical significance, *P* < 0.0033 using the Mann–Whitney *U*-Test. PFG, posterior frontal grey matter; AFG, anterior frontal grey matter; AFWM, anterior frontal white matter; TG, temporal grey matter; TWM, temporal white matter; PG, parietal grey matter; PWM, parietal white matter; SN, substantia nigra; PONS, pons; CAUD, caudate; PUT, putamen; GP, globus pallidus; STN, subthalamic nucleus; DENT, dentate nucleus; CWM, cerebellar white matter.

**Figure 2:**
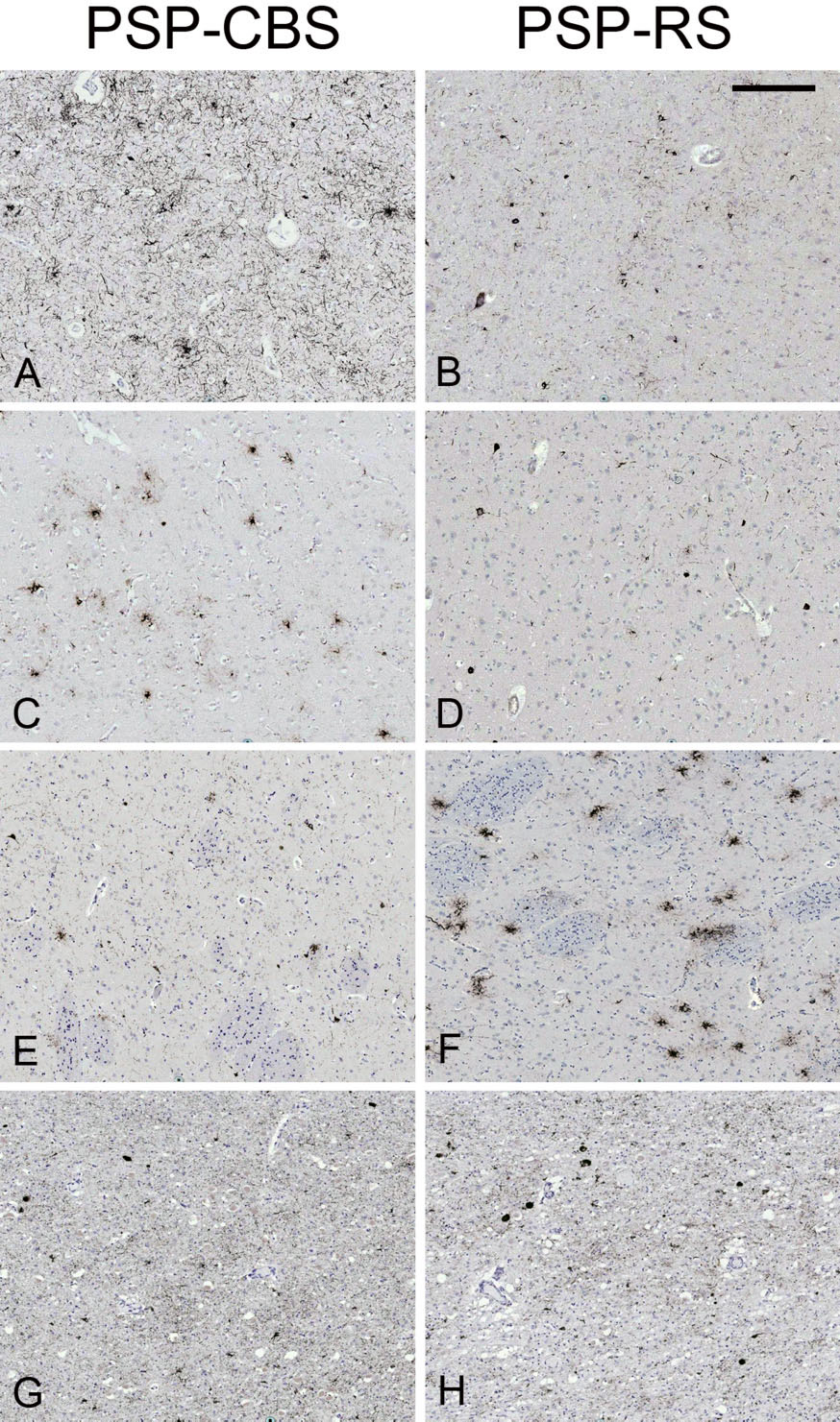
Tau immunohistochemistry in selected brain regions of the two most representative cases. PSP-CBS has significantly greater tau load in the posterior frontal (A) and anterior frontal grey matter (C) when compared with PSP-RS (B, D). Regional tau load in the caudate and subthalamic nucleus are greater in PSP-RS (F, H) than in PSP-CBS (E, G). Median tau load values. Posterior frontal grey matter: (A) PSP-CBS: 1.09; (B) PSP-RS: 0.05; Anterior frontal grey matter: (C) PSP-CBS: 0.18; (D) PSP-RS: 0.04; Caudate: (E) PSP-CBS: 0.11; (F) PSP-RS: 1.06; Subthalamic nucleus: (G) PSP-CBS: 0.05; (H) PSP-RS: 0.29. Tau load for each region, that is, ‘regional’ tau load was expressed as percentage (areal fraction × 100%); ‘areal fraction’, which was computed by Image Pro, was defined by a ratio of the tau-positive immunoreactive pixels to the total number of pixels of the whole field. AT8 immunohistochemistry, bar in panel B represents 225 microns in all the panels.

The median ‘regional’ tau load in the posterior frontal cortical grey matter (PSP-CBS: 0.59; PSP-RS: 0.05), anterior frontal cortical grey matter (PSP-CBS: 0.06; PSP-RS: 0.03) and parietal subcortical white matter (PSP-CBS: 0.06; PSP-RS: 0.01) was significantly greater in PSP-CBS than in PSP-RS (*P* < 0.0033 in all). The median ‘regional’ tau load in the caudate (PSP-CBS: 0.14; PSP-RS: 0.49; *P* < 0.001), STN (PSP-CBS: 0.21; PSP-RS: 0.40; *P* < 0.001) and cerebellar white matter (PSP-CBS: 0.02; PSP-RS: 0.06; *P* = 0.007 with borderline significance) was greater in the PSP-RS than in PSP-CBS (Figures 1 and 2).

The presence of delayed initiation of horizontal saccades in PSP-CBS had a moderate correlation with an increased total parietal ‘tau load’ (Spearman's correlation coefficient = 0.59; *P* < 0.001). However, other cortical features such as cortical sensory loss, alien limb phenomenon or hemi-sensory neglect did not correlate with the parietal ‘tau load’ (*P* > 0.05) or other ‘regional tau load’.

#### ‘Total’, ‘cortical’ and ‘basal ganglia’ tau load (Figure 3)

**Figure 3:**
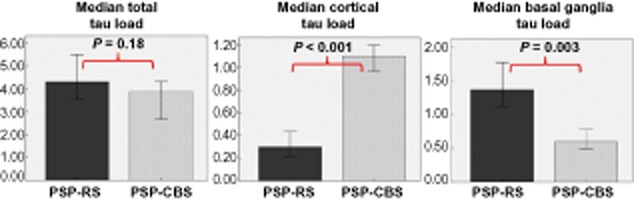
The median ‘total tau load’ between the PSP-CBS and PSP-RS groups are the same. However, the PSP-CBS group has greater median ‘cortical tau load’ and less ‘basal ganglia tau load’ than the PSP-RS group (Mann–Whitney *U*-Test). ‘Cortical tau load’ is the sum of regional tau load of posterior frontal grey matter, anterior frontal grey and white matter, temporal grey and white matter, parietal grey and white matter. ‘Basal ganglia tau load’ is the sum of regional tau load of caudate, putamen, globus pallidus and subthalamic nucleus. ‘Total tau load’ is the sum of regional tau load of all 15 brain regions. Error bars represent 95% confidence interval.

There was no difference in ‘total’ tau load between the PSP-CBS and PSP-RS groups (*P* = 0.176, Figure 3). However, PSP-CBS had an increased ‘cortical’ tau load when compared with PSP-RS (*P* < 0.001); and the ‘basal ganglia’ tau load was greater in PSP-RS than in PSP-CBS (*P* = 0.003).

In five PSP-CBS cases, the half brains examined were contralateral to the side with the more predominant clinical symptoms and signs. The median ‘total’ and ‘cortical’ tau load were numerically, but not statistically, greater in these five cases (total tau load = 5.3; cortical tau load = 1.4) compared with the remaining PSP-CBS cases (total tau load = 4.0; cortical tau load = 1.0).

#### Tau-positive cellular lesions

In the posterior frontal cortical grey matter, all types of tau lesions were more numerous in PSP-CBS than in PSP-RS (NFTs, TAs, CBs and NTs: *P* < 0.001; PreTs: *P* = 0.005). In the anterior frontal grey matter, there were numerically, but not statistically, more NFTs, CBs and NTs in PSP-CBS than in PSP-RS (*P* > 0.01 in all). In the caudate, there were more TAs, NTs, and NFTs, in PSP-RS than in PSP-CBS (TAs and NTs: *P* < 0.001, NFTs: *P* = 0.01).

#### Neuronal loss

**Figure 4:**
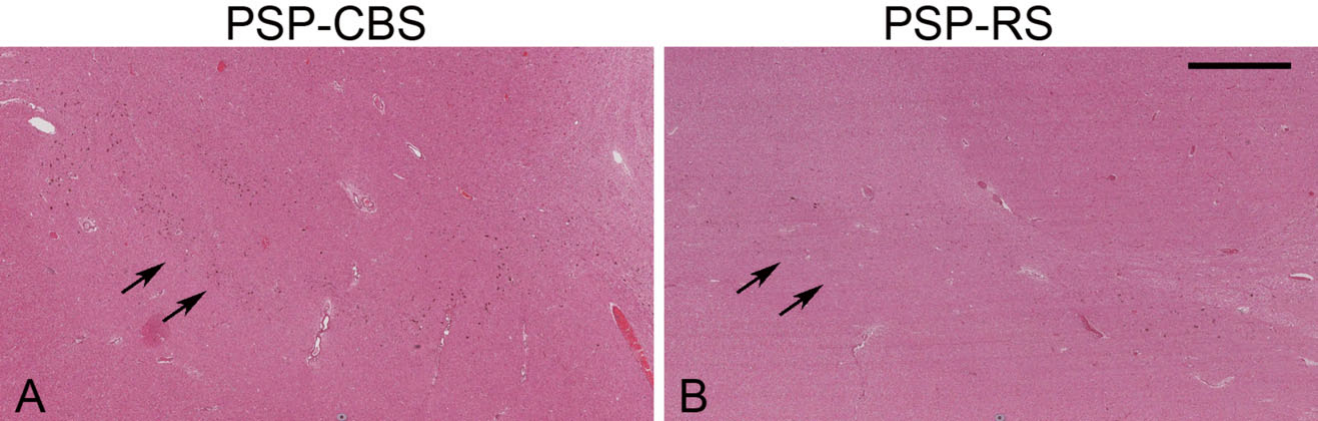
PSP-RS (B) has greater degree of neuronal loss in the ventrolateral (arrows) and dorsolateral substantia nigra than in PSP-CBS (A). Haematoxylin and eosin method, bar in panel B represents 1135 microns in both panels.

In the STN, the median semi-quantitative rating score for neuronal loss was moderate (grade 2) and there was no difference between the two groups (χ^2^; *P* ≥ 0.05). In the SN, neuronal loss was more severe in the dorsolateral (χ^2^; *P* = 0.033) and ventrolateral (χ^2^; *P* = 0.018) subregions in PSP-RS than in PSP-CBS (Figure 4).

#### CST involvement

**Figure 5:**
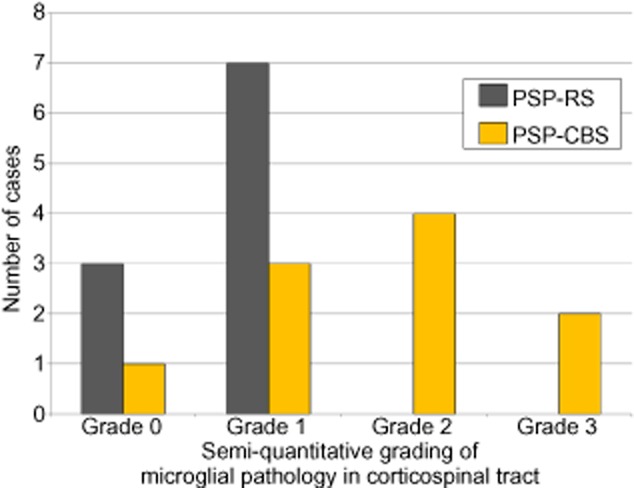
A more severe microglial pathology in the corticospinal tract was identified in PSP-CBS when compared with PSP-RS (χ^2^; *P* = 0.035). A semi-quantitative grading scale was used to characterize the severity of microglial pathology; grade 0 = baseline microglial population, grade 1 = mild microglial pathology; grade 2 = moderate microglial pathology; grade 3 = severe microglial pathology.

There was a more severe microglial response in the CST in PSP-CBS, ranged from mild to severe, than in PSP-RS, in which CST involvement was very mild (χ^2^; *P* = 0.035) (Figure 5).

#### Additional pathological findings

The CERAD Aβ plaque score ranged from ‘absent’ to ‘sparse’, except for two PSP-CBS and one PSP-RS cases, which had a ‘moderate’ score 27. Small vessel cerebrovascular disease was noted in one PSP-CBS and one PSP-RS case. Other additional pathological findings are summarized in Table 4.

**Table 4:** Additional pathological findings of PSP-CBS and PSP-RS groups

	PSP-CBS	PSP-RS
CERAD neuritic plaque score		
Negative	1	1
Infrequent	7	8
Moderate	2 (cases 5 & 7)	1 (case 15)
Frequent	0	0
Tau-positive AGD	4 (cases 3, 5, 8, 9)	4 (cases 11, 14, 16, 18)
Incidental Lewy body disease	1 (Braak stage 3)	0
Cerebrovascular pathology	1 (mild)	1 (severe, case 19)
Cerebral amyloid angiopathy	1 (mild)	0

AGD, argyrophilic grain disease; CERAD, Consortium to Establish a Registry for Alzheimer's disease.

### Tau biochemistry and haplotype analysis

**Figure 6:**
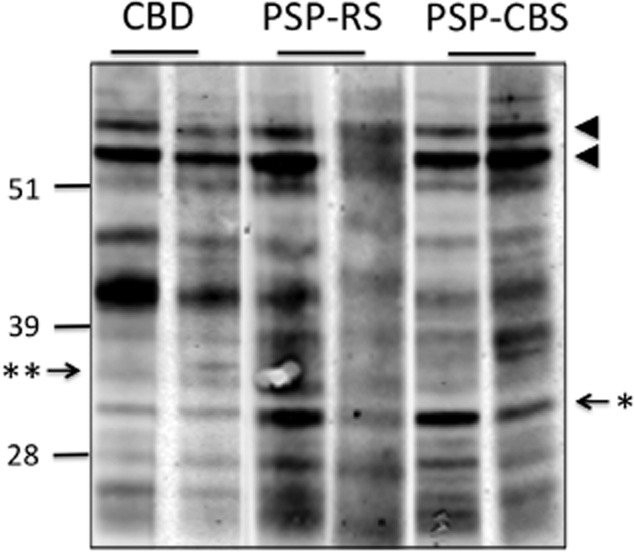
Western blot analysis of sarkosyl-insoluble tau from frontal cortex homogenates of CBD-CBS, PSP-CBS and PSP-RS. The characteristic doublet of predominant 4R-tau pathology was observed in both CBD and PSP cases (arrowheads). CBD-CBS cases have a lower molecular weight doublet consisting of proteolytic fragments at approximately 37 kDa (**); whereas PSP-RS and PSP-CBS cases have a single band at approximately 33 kDa (*). Numbers on the left indicate positions of molecular weight markers (kDa).

Western blots of the sarkosyl-insoluble tau fractions from the frontal cortical homogenates showed the characteristic doublet at 64 and 68 kDa indicating predominant 4R tau in PSP-CBS, PSP-RS and CBD-CBS cases (Figure 6). Our PSP-CBS and PSP-RS cases showed a single band at approximately 33 kDa, whereas the CBD-CBS cases had a doublet at approximately 37 kDa (Figure 6), which are consistent with previous findings on the molecular differences in the low molecular weight proteolytic fragments between CBD and PSP 14,40. There was no significant association between H1/H1 or H1/H2 genotype with either of the PSP subgroups (χ^2^ test; *P* = 0.21, Table 1).

## Discussion

We compared the morphological, biochemical and genetic characteristics of 10 clinically well-characterized PSP-CBS cases and 10 age- and disease duration-matched PSP-RS controls. Irrespective of the clinical presentation, all our cases met established neuropathological diagnostic criteria of PSP with the presence of 4R tau-positive neuronal and glial inclusions, including TAs in a characteristic distribution 20–22. Our biochemical studies of sarcosyl insoluble tau also confirmed 4R tau as the main protein species in both PSP-RS and PSP-CBS groups and the presence of a smaller, faster migrating carboxy-terminal fragment as previously reported in PSP 40,41. We found no difference in the distribution of the H1/H1 and H1/H2 haplotypes between the two PSP groups.

We used morphometry to compare tau load, defined as the sum of all tau positive lesions in the brain regions studied, between the two PSP groups and to determine the contribution of different neuronal and glial lesions to the tau pathology. A previous study at the Mayo Clinic established that PSP-CBS was associated with a greater tau burden in the mid-frontal and inferior-parietal cortices than in PSP-RS 12. Here, by assessing a greater number of cases and more brain regions, we validated their findings by showing increased tau load in the cortical regions predominantly in the posterior frontal grey matter, anterior frontal grey matter and parietal white matter in PSP-CBS. We also extended the quantitative tau assessment to other non-cortical regions which enabled us to identify a reduced tau load in the caudate, STN and cerebellar white matter in the CBS variant. It is noteworthy that the increased cortical tau load is compensated by the reduced basal ganglia tau load in PSP-CBS resulting in the total tau load, determined as the sum of the all regional tau load, being similar in the two PSP groups.

Increased cortical tau pathology has also been documented in PSP variants with cortical features including ‘atypical’ PSP with progressive apraxia of speech and non-fluent aphasia 42, PSP-bvFTD 11,43, and, together with PSP-CBS, these clinical phenotypes are collectively referred as the ‘cortical’ PSP variants 44. On the other hand, PSP-P and PSP-PAGF, which are considered as the ‘brainstem’ variants of PSP, have less severe overall tau pathology when compared with PSP-RS 5,6. Interestingly, these ‘brainstem’ variants are associated with a more benign disease course and a longer disease duration compared with the classical PSP-RS 5,6,30; whereas the disease duration of our PSP-CBS group was similar to that of PSP-RS group previously reported 2. We speculate that the total tau pathology may inversely correlate with the disease duration in PSP variants and while the ‘brainstem’ PSP variant appears to be a more ‘benign’ form of PSP, the ‘cortical’ PSP variant represents a deviation from the classical presentation determined by a shift of tau pathology from the basal ganglia to the cerebral cortex. By selecting only cases with limited Alzheimer-type NFT pathology and assessing coexisting secondary pathologies, it is clear that the clinical presentation in our PSP-CBS cases was closely associated with the topographical severity of tau pathology which could not otherwise be explained by secondary pathologies. We also demonstrated that the regional differences in tau load between the two PSP groups were contributed by neuronal and glial lesions characteristic of PSP pathology rather than Alzheimer-related tau pathology 45.

A recent detailed clinicopathological study from the Mayo Clinic compared the characteristics of CBS and RS clinical phenotypes in pathologically confirmed CBD cases 14. Their study on CBD also demonstrated significant differences in the topographical severity of tau pathology between the two CBD subtypes, which correlated with the different clinical presentations. Similar to the findings in our PSP-CBS cases, their study showed that the CBD-CBS cases had more severe tau deposition in the cortical regions and less severe tau pathology in the lower brainstem and cerebellum when compared with the CBD-RS cases. However, total tau load and the contribution by different neuronal and glial lesions to the tau pathology were not assessed 14.

The STN is one of the regions characteristically targeted by the PSP disease process 20,46 while this nucleus is better preserved in cases with pathologically confirmed CBD. In our PSP-CBS cases, the atrophy and neuronal loss in the STN was as severe as in the PSP-RS cases, despite the regional tau load of the STN being less in PSP-CBS than in PSP-RS, indicating that glial, rather than neuronal tau, might have significantly contributed to the differences in tau load. It is noteworthy that a relatively milder tau pathology in the STN has been documented in other PSP variants such as ‘atypical’ PSP with progressive apraxia of speech and non-fluent aphasia 42.

In PSP, cell loss in the SN affects both the pigmented neurones of the pars compacta and non-pigmented neurones in the pars reticulata, whereas neurones in the medial nigra are relatively preserved 47. In the present study, neuronal loss was less severe in the ventrolateral and dorsolateral subregions in PSP-CBS when compared with PSP-RS (Figure 4). This regional difference may, in part, influence the clinical features due to the resulting selective damage to the dopaminergic and GABAergic neuronal nigral projections 47.

Pyramidal signs were documented in half of our PSP-CBS cases, but they were absent in our PSP-RS cohort. Pyramidal signs are relatively uncommon in PSP and in one series they were present in only one fifth of all pathologically confirmed PSP cases 2. On the other hand, 60% of pathologically confirmed CBD cases had pyramidal signs 48. In CBD, the pathological involvement of the primary motor cortex including loss of Betz cells is a common finding, explaining the presence of pyramidal signs 44,48. The common occurrence of pyramidal signs in our PSP-CBS cohort can be explained by the abundant tau pathology in the primary motor cortex, which was 12-fold greater in PSP-CBS than in PSP-RS. There was also more severe microglial pathology in the CST in PSP-CBS than in PSP-RS. CST degeneration and significant tau pathology in the motor cortex are also prominent in a group of ‘atypical’ PSP cases reported in the literature which sometimes clinically present with CBS 44,49; however, whether these cases should be classified as PSP has recently been questioned 50.

All PSP-CBS cases had received a final clinical diagnosis of CBS and they all had markedly asymmetrical cortical and extrapyramidal features, including unilateral limb clumsiness with a progressively maladroit and functionally useless hand. In the past, the presence of marked asymmetrical clinical signs would exclude the clinical diagnosis of PSP, but this concept has been challenged in recent years with the findings of clinicopathological series confirming asymmetrical presentations in some PSP variants 2,17. Previously, a *post mortem* report of a Japanese patient who had focal limb dystonia and levitation revealed significantly more tau pathologies in the frontal cortices, basal ganglia and brain stem in the contralateral half brain than the ipsilateral half brain 51. In our PSP-CBS cohort, there was numerically greater total tau load and cortical tau load in five cases where the contralateral half brain was available for evaluation when compared with the other five cases where ipsilateral half brain was examined. However, we cannot conclude if the tau load is greater in the clinically more manifested hemisphere within an individual as only half brains were used. Asymmetrical limb apraxia and delayed initiation of horizontal saccades are clinical features suggestive of underlying parietal lobe dysfunction and are characteristic features of CBS 52. We found that regional tau load in the parietal white matter was 5-fold greater in PSP-CBS than in PSP-RS and that PSP-CBS patients who had delayed initiation of horizontal saccades also had greater regional tau load in the parietal cortex and white matter.

Three patients had delayed initiation of horizontal saccades and, interestingly, half of them also had VSGP in the late stage of the illness, with involvement of downgaze, a diagnostic prerequisite for the diagnosis of PSP-RS 53,54. VSGP is rare in CBD with classical CBS presentation (CBD-CBS), and was noted in only 18% of cases in the Mayo Clinic series 14 and was not observed in the QSBB series 17. Six PSP-CBS patients had recurrent falls in the first year of their illness, whereas early falls were less frequent in CBD-CBS cases and were recorded in only 20% and 18% in the QSBB and Mayo Clinic series, respectively 14,17. We postulate that early postural instability, falls and supranuclear downgaze palsy in patients with CBS are clinical clues which when present, suggest an underlying PSP pathology even though there are also signs of CBS. Nevertheless, three cases in our PSP-CBS group (cases 2, 7, 10) had a pure CBS presentation throughout the disease course and did not have any tell-tale signs of PSP. This is in concordance with our experience based on clinicopathological evaluation of cases in the QSBB that some pathologically confirmed PSP and CBD cases present with a pure clinical syndrome such as CBS or RS irrespective of the underlying pathology, whereas some cases manifest overlap clinical features such as RS or CBS at the same time and occasionally, the clinical syndromes temporally evolve from one to another throughout the disease course as previously described by Kertesz *et al*. 9.

Data from transgenic animal studies indicate that soluble rather than fully aggregated tau species may ultimately be responsible for neuronal degeneration and cell death 55. However, the findings in this study support the notion that neuronal and glial inclusions composed of fibrillar pathological tau are useful and clinically valid pathological markers of the underlying neurodegenerative process. We have provided comprehensive evidence that the topographical severity of tau pathology in PSP is closely associated with its clinical manifestation 5,6,11,30,42. This is comparable to the findings in Alzheimer's disease where cognitive deficit shows a far better correlation with tau lesions than with Aβ plaques 56 as well as in other primary tauopathies 44,57. A better understanding of the factors that influence the selective pathological vulnerability in different PSP variants will provide further insights into the neurodegenerative process underlying tauopathies.
